# Reply: Forkhead box P3-positive regulatory T cells in immune surveillance and cancer

**DOI:** 10.1038/sj.bjc.6603940

**Published:** 2007-08-14

**Authors:** G Betts, A Godkin, A Gallimore

**Affiliations:** 1 Medical Biochemistry and Immunology, Henry Wellcome Building, Heath Park, Cardiff CF14 4XN, Wales, UK

**Sir,**

Carcinogen-induced tumours in intact mice exhibit a substantial enrichment of CD4^+^FOXP3^+^ regulatory T cells (Tregs) (Betts *et al*, 2007) and many forms of malignant disease are associated with an expansion of circulating Tregs in patients (reviewed in Betts *et al*, 2006). Galon *et al* (2006) demonstrated recently that the type, density and location of T cells within colorectal tumours predicted clinical outcome. In contrast, Tregs have been shown to increase with progression of malignant disease and to correlate negatively with prognosis (Curiel *et al*, 2004; Wolf *et al*, 2005). Definitive studies need to be performed to establish the relative contribution of the accumulation of Tregs among tumour-infiltrating lymphocytes to prognosis since it is currently unclear if an early accumulation of Tregs into the tumour environment allows disease progression. Future studies should include a retrospective analysis of Treg infiltration of tumours removed from patients to establish whether patients with early disease and a relatively large infiltration of Tregs had a worse prognosis than predicted by conventional histopathological scoring and conversely, whether those with advanced disease but a relatively low infiltration of Treg had a better prognosis than predicted.

Evidence collected from mice suggests that depletion of Tregs enhances immunosurveillance of tumours and uncovers new responses to tumour antigens in patients (Curiel *et al*, 2004; Clarke *et al*, 2006). Hence, removal of Tregs may represent a component of future strategies to trigger immune-mediated elimination of tumour tissue. Nishikawa *et al* (2003) demonstrated that vaccination with self-antigen expanded Treg with enhanced FOXP3 expression and suppressive capacity. This highlights the importance of co-depleting Treg not only to boost tumour-specific immune responses but also to suppress expansion of Treg with shared antigen specificity.

The optimal vaccination/depletion strategy needs to be established by defining the impact of preoperative chemotherapy and surgery on the development of antitumour immune responses. Surgical removal of malignant disease is likely to remove the bulk of Treg TIL and reduce the production of suppressive signalling networks, which would undoubtedly improve the likelihood of successful antitumour immune responses to clear residual malignant cells. However, the impact of major surgery on the capacity of the immune response needs to be established. It is also important to weigh up the benefit of postoperative immunotherapy and adjuvant chemotherapy. Chemo/radiotherapy might reduce the capacity of the immune system to mount antitumour immunity. In light of conflicting reports describing the ability of certain treatments to deplete Treg efficiently (Attia *et al*, 2005; Dannull *et al*, 2005), conventional chemotherapeutic drugs that are proven to deplete Treg might be the best starting point to develop strategies to co-vaccinate and deplete Treg. Tregs have been shown to be highly susceptible to cyclophosphamide (Ghiringhelli *et al*, 2004) and fludaribine (Beyer *et al*, 2005) and previous work by North (1982) has indicated that vaccination with tumour antigens might go hand-in-hand with chemotherapy to deplete Treg and promote antitumour immunity.
